# Cause and Prevention of Consumption—Is It a Curable Disease?*Read before Georgia Medical Association, April, 1897.

**Published:** 1897-07

**Authors:** J. S. Todd

**Affiliations:** Atlanta, Ga., Professor Therapeutics and Materia Medica, Atlanta Medical College; ex-President of the Association, etc.


### CAUSE AND PREVENTION OF CONSUMPTION.—IS IT A CURABLE DISEASE?*

BY J. S. TODD, M.D., ATLANTA, GA.,
Professor Therapeutics and Materia Medica, Atlanta Medical College; ex-President of the Association, etc.

Consumption is a contagious and infectious disease, preventable and curable. It is generally defined as an infectious disease only. The two words contagion and infection are often used synonymously, and the confusion which has arisen therefrom is not settled. Therefore it best serves my purpose to say it is both, at the risk of being scientifically inexact.

That it is contagious and infectious is proven by the fact that it can be inoculated; that the air of an apartment or locality can be so highly impregnated with the specific cause that its inhalation will give rise to the disease; that in a dried state—as on clothing—it can be transported and not lose its virulence, as is so often exemplified by the frequency of washerwomen contracting the disease from those infected.

That the disease is caused by a specific organism—the tubercle bacillus of Koch—a germ *sui generis*—is now almost universally admitted. His demonstrations have stood all the tests and fulfill every assertion; the specific organism is always present in every case.

In the language of Dr. Ainsworth, of Brooklyn, N. Y., in vol. LXX., No. 3, *Medical News*—"It has satisfied every condition of proof that can be demanded, in that it conforms to the requirements of the formulated proposition to demonstrate that a given disease is caused by a specific organism: 1st. It is present in all cases of the disease and in such distribution as will explain the lesions. 2d. It can be isolated in pure culture. 3d. The disease is reproduced by inoculation with the isolated organisms." More convincing proof could not be procured, nor reasonably demanded.

The disease is not hereditary; that is, a child is seldom born with it, so rarely that the exception proves the rule.

* Read before Georgia Medical Association, April, 1897.

The children of parents, both consumptive, need not necessarily have the affection; indeed, such progeny, if isolated, or passing their lives in a locality where there were no cases of the disease, would never contract or develop it. They are more predisposed to the malady than the children of the healthy, *if exposed*; they inherit a soil that furnishes ready and rich pabulum and favorable conditions for its growth and development, but the germ—the cause—is the product of contaminated environment, not a development of inherited tubercle. But isolation or absolutely pure air is not necessary to at least greatly reduce the prevalence of phthisis now that we know the cause.

Another fact brought out by knowing the cause is that none of us are immune. It can be contracted, and it is a sad fact often the case that those with no hereditary predisposition, individuals with no history of tuberculosis for generations, succumb to the malady; and whole communities which but a short while ago were celebrated as health resorts for consumptives, are now peopled by a rapidly-disappearing population, victims of this contagious disease.

As illustrations of the latter assertions, Monaco, in France, and the greater prevalence of the affection in Colorado, Florida, Southern California, or wherever consumptives congregate, are unanswerable proofs of its contagious and infectious character. It was practically unknown among the negroes when they were slaves; now it is so common with them that it has failed to excite comment. It is generally stated by the best informed writers on this disease, that one-seventh of all the deaths are attributable directly or indirectly to the ravages of this minute germ. No country or people are totally exempt: all living animals, even the cold-blooded, are inoculable with it.

We dread cholera, plague, smallpox, diphtheria, yellow fever, but none of these are more contagious than consumption; all put together do not cause half the mortality; each of the first named are only occasionally epidemic with us, and are under strict police surveillance, guided by health authorities, to prevent their spread; the latter has become almost a household disease in many localities; no community is exempt, and still we raise no warning voice. The people think the disease is entirely hereditary, therefore not preventable. But I am thankful that the great city of New York has awakened to the importance of this subject, and after a campaign of information lasting several years, has at last declared consumption a communicable disease, and made it incumbent on physicians to report all cases to the health authorities. Their circular of information is so replete with information that I incorporate it entire in this article. It is published in English, German, Italian and Hebrew, and scattered broadcast—verily "bread upon the waters." I ask your careful attention to its reading:

HEALTH DEPARTMENT,
CRIMINAL COURT BUILDING, NEW YORK CITY.

Consumption is a disease which can be taken from others, and is not simply caused by colds. A cold may make it easier to take the disease. It is usually caused by germs which enter the body with the air breathed.

The matter which consumptives cough or spit up contains these germs in great numbers—frequently millions are discharged in a single day. This matter, spit upon the floor, wall or elsewhere, is apt to dry, become pulverized, and float in the air as dust. The dust contains the germs, and thus they enter the body with the air breathed. The breath of a consumptive does not contain the germs, and will not produce the disease. A well person catches the disease from a consumptive only by in some way taking in the matter coughed up by the consumptive.

Consumption can often be cured if its nature is recognized early and proper means are taken for its treatment. *In a majority of cases it is not a fatal disease.*

It is not dangerous for other persons to live with a consumptive, if the matter coughed up by the consumptive is at once destroyed. This matter should not be spit upon the floor, carpet, stove, wall or street, or anywhere except into a cup kept for that purpose. The cup should contain water, so that the matter may not dry, and should be emptied into the closet at least twice a day and carefully washed with hot water. Great care should be taken by a consumptive that his hands, face and clothing do not become soiled with the matter coughed up. If they do become soiled, they should at once be washed with hot soap and water. When consumptives are away from home, the matter coughed up may be received on cloths, which should be at once burned on returning home. If handkerchiefs are used (worthless cloths which can be burned are much better) they should be boiled in water by themselves before being washed.

It is better for a consumptive to sleep alone, and his bed-clothing and personal clothing should be boiled and washed separately from the clothing belonging to other people.

Whenever a person is thought to be suffering from consumption, the name and address should be sent at once to the Health Department, on a postal card, with a statement of this fact. A medical inspector from the Health Department will then call and examine the person to see if he has consumption, providing he has no physician, and, if necessary, will give proper directions to prevent others from catching the disease.

Frequently a person suffering from consumption may not only do his usual work without giving the disease to others, but may also get well, if the matter coughed up is properly destroyed.

Rooms that have been occupied by consumptives should be thoroughly cleaned, scrubbed, whitewashed, painted or papered before they are again occupied. Carpets, rugs, bedding, etc., from rooms which have been occupied by consumptives, should be disinfected. The Health Department should be notified, when they will be sent for, disinfected and returned to the owner free of charge, or, if he so desires, they will be destroyed.

By order of the Board of Health.

CHARLES G. WILSON, President.

EMMONS CLARK, Secretary.

Comment is unnecessary; commendation is all I have to bestow. There has been a very perceptible diminution of consumption in New York in the past five years.

Is the disease curable? My object being to disseminate the truth, not to magnify myself, I let Vaughan, of Ann Arbor, answer, and I beg no pardon for having him say for me what I believe, especially as he does it so much better, and it should and doubtless does, carry with it so much more authority.

"In 1882 Baumgarten wrote, in sum and substance, as follows: 'Tuberculosis is in and of itself not so bad a disease. We do not believe that it possesses, as a constant property, the considerable local malignancy which Friedlander believes he must still unconditionally attribute to it. When we see individuals, who as children had thickened tuberculosis, cervical glands or tuberculous disease of the joints, develop into strongest and handsomest men; when we see people, who in childhood had Pott's disease, which is always due to the tubercular caries of the vertebræ, live for years with this deformity, and reach advanced age; with such observations as these so frequently before us, we must conclude that tuberculosis is not so deadly as is usually supposed.'

"Tuberculosis, so long as it remains an unmixed infection, is not a deadly disease, and I believe it to be, in this stage, one of the most easily curable of the bacterial diseases. Except in the form of acute miliary and meningeal tuberculosis, it is but seldom the direct cause of death. However, as soon as it becomes a mixed infection, and is something more than tuberculosis, it then takes rank as the most fatal of all diseases now afflicting mankind. In combating this disease, there are two points of attack, and if the wisdom and skill of the profession be directed to these, the death-rate from tuberculosis will be much reduced in the near future. The first and most important method of dealing with this disease consists of the application of preventive measures. The second, the one with which I am now concerned, consists of the treatment of the disease while it remains an unmixed infection. When this is done, tuberculosis will cease to be 'the terror of the laity and the cross of the physician.' In order to accomplish this work, however, we must put aside certain dogmas, which we have so long mistaken for facts that their shadows frighten us. We must be convinced that as an unmixed infection it is curable. We must be able to recognize the disease while it remains an unmixed infection. We must cease regarding certain conditions as evidence of existence of indefinite, imaginary, so-called 'pre-tubercular state,' and must learn to look upon these conditions as proof of the actual existence of tuberculosis. I will now give attention to these points, and in doing so I will consider them quite apart from the claims which I may make for nuclein, or any other agent employed for curative purposes. One-seventh of all men die of tuberculosis, and one-third of all men have the disease. The first part of the preceding statement is shown to be true by the mortality statistics of all civilized countries. The second part of the same statement is shown to be true by the records of autopsies in various parts of the world. Massini found evidence of tuberculosis, as shown by scars, indurations, cheesy and chalky foci, in eighty-nine out of two hundred and twenty-eight autopsies made upon persons who had died of diseases other than tuberculosis at the Basle Hospital. Muller found in the Munich Pathological Institute that tuberculosis caused 29.4 per cent. of deaths in adults, and 30 per cent. in children, and that 11.8 per cent. more of the children had tuberculosis. Bollinger's researches confirm these statements. Baumgarten stated that from one-fourth to one-third of all the bodies sectioned show tubercular lesions. Ilneyrat found tuberculosis in 31.4 per cent. of the children examined. Lundonzy reports that 30.4 per cent. of the children brought to autopsy are tuberculous. Babes found tubercular deposits in half the children dying from all diseases at Bucharist; in 1887, he sectioned 93 children, 65 of whom had tubercular glands. Haitmel claims one-third of the children examined at L'Hospiel des Enfants Assistes show tubercular lesions. Simonds, Schwer and Boltz state that autopsies of the Anatomical Institute at Kiel, of 781 children, aged from one to five years, show tubercular lesions in 230; of 228, between five and ten years, 78 were tubercular; of 162, between ten and fifteen years, 56. Wolff states that autopsy reveals either recent or old tubercular lesions in from 40 to 45 per cent. in adults, and in 60 to 70 per cent. of children; also that 30 per cent. of adults, and 40 per cent. of children have latent tuberculosis. Schlenker found in one hundred consecutive autopsies at Zurich, 66 bodies tubercular; 35 of these had died of tuberculosis; in four others the disease was marked, but had not caused death; while in twenty-seven it was latent, and had not been suspected during life. In his examinations no lesions were pronounced tubercular unless it was evident and characteristic to the unaided eye. He points out the fact that had the indurations been examined microscopically, the percentage of cases of latent tuberculosis would have been increased. According to Adossides, of 4,815 bodies sectioned at Halle, from 1882 to the middle of 1893, 1,066 showed gross tubercular lesions. These, and other statistics of like import, justify the assertion that about one-third of all men are, at some period of life, infected with tuberculosis. Now, if one-third be infected, and one-seventh die of the disease, it must be that the difference between one-third and one-seventh, a little more than one-fifth, are tubercular, and do not die of the disease. Of the sixty-three millions of people living in the United States in 1890, nine millions have died or will die of tuberculosis, while twenty-one millions have been or will be infected with the bacillus tuberculosis. Does not this demonstrate that tuberculosis does not possess the malignancy attributed to it? The fact that one may be infected with tuberculosis, and such infection remain localized for many years is shown by the study of cases of lupus. The study of instances of accidental inoculation with the bacillus of tuberculosis in wounds, sometimes known as cadaver tuberculosis, tends to similar results. Tuberculous joints often heal, and the child afflicted in this way may develop into the most robust adult. The evidence that tuberculosis of the peritoneum occasionally undergoes a spontaneous cure, and frequently heals after laparotomy, is no longer questionable."*

Tuberculosis is phthisis pure and simple; caseous pneumonia is tuberculosis of the lungs unmixed; consumption—phthisis—is mixed tuberculosis, tuberculosis plus something else; each has the same origin—the tubercle; one, the spring, the fountain-head; the other, the mighty swift, muddy, and irresistible river.

I have no mixed prescription to give as a cure for consumption, I have no fixed prescription to give as a cure for consumption, die, because we do not recognize the disease early, I firmly assert. We should seldom diagnose a case as consumption, even in the latter stage, when all the general train of symptoms, physical and otherwise, are present, positively, as undoubtedly such, until we can and do find the bacillus; nor, on the other hand, should we conclude that we have made a scientific diagnosis—a diagnosis by exclusion— in any case, with the least suspicious symptoms, until we have failed to find it, after thorough and repeated microscopic search. While the figures given by Vaughan, as to the prevalence of tuberculosis are far below those of others, and granting that for these United States they are still too high, we may even then look with horror upon the picture.

Reducing this statement, that one-third of those now living in our own country have or will contract the disease, to one-fifth, and the mortality of the disease as the cause of all deaths, to one-tenth, and what do we read? Listen! Of the 70,000,000 people in these

*Medical News, Vol. LXX., No. 10.

United States, 14,000,000 will contract tuberculosis, if preventive measures are untaught, unknown, and unheeded; 7,000,000 will fill premature graves, to gratify this insatiate monster, the bane of humanity, if we fail to diagnose it in its incipiency.

Truly the harvest field for good is vast; shall the laborers be few?

To prevent is better than to cure, and while I would not detract from the luster and fame that belongs to discoverers of the specific for malaria, quinine, and thank God for those bright stars, Couventou and Pellitere; yet all concede, that in the medical galaxy the star of greatest magnitude, surrounded by its halo of imperishable glory, is named Jenner the Preventer.

